# Adaptive Laboratory Evolution of Native *Torulaspora delbrueckii* YCPUC10 With Enhanced Ethanol Resistance and Evaluation in Co-inoculated Fermentation

**DOI:** 10.3389/fmicb.2020.595023

**Published:** 2020-12-21

**Authors:** Daniela Catrileo, Andrea Acuña-Fontecilla, Liliana Godoy

**Affiliations:** Departamento de Fruticultura y Enología, Facultad de Agronomía e Ingeniería Forestal, Pontificia Universidad Católica de Chile, Santiago, Chile

**Keywords:** potassium metabisulfite resistance, adaptive evolution, *Torulaspora delbrueckii*, ethanol resistance, non-*Saccharomyces* yeasts

## Abstract

*Torulaspora delbrueckii* is a yeast species typically present in the early stages of the fermentation process. *T. delbrueckii* positively modifies the aromatic properties of wines. However, its contribution to the final quality of the wine is restricted by its low tolerance to ethanol. *T. delbrueckii* is capable of fermenting and tolerating an ethanol concentration ranging from 7.4% (v/v) to slightly higher than 9% (v/v). For this reason, it cannot complete fermentation, when alcohol reach levels higher than 12% (v/v), limiting their use in the industry. The objective of this work was to obtain new variants of *T. delbrueckii* with improved resistance to ethanol through adaptive laboratory evolution. Variants capable of tolerating ethanol levels of 11.5% (v/v) were obtained. These presented improved kinetic parameters, and additionally showed an increase in resistance to SO_2_ in ethanol compared to the original strain. Co-inoculated fermentations were performed with the original strain (FTd/Sc) and with the evolved strain (FTdF/Sc), in addition to a control fermentation using only *Saccharomyces cerevisiae* EC1118 (FSc). The results obtained show that FTdF/Sc present higher levels of 2-Ethylhexanol, compared to FTd/Sc and FSc. Furthermore, FTdF/Sc presents higher levels of total alcohols, total aldehydes, total phenolic derivatives, and total sulfur compounds with significant differences with FSc. These results provide a *T. delbrueckii* YCPUC10-F yeast with higher resistance to ethanol, which can be present throughout the fermentation process and be used in co-inoculated fermentations. This would positively impact the performance of *T. delbrueckii* by allowing it to be present not only in the early stages of fermentation but to remain until the end of fermentation.

## Introduction

Wine fermentation is a complex process involving different microorganisms, such as yeasts, bacteria, and filamentous fungi. Although *Saccharomyces cerevisiae* is responsible for alcoholic fermentation, other yeast species (Fleet et al., 1984) also participate, and are present in early stages of fermentation due to their low fermentative capacity, being rapidly outcompeted by *S. cerevisiae*. However, it has been reported that the metabolic activity of non-*Saccharomyces* yeasts plays a fundamental role in the final quality of wine (Ciani and Maccarelli, 1997; Jolly et al., 2006, 2013; Ciani et al., 2010; González-Royo et al., 2015; Godoy et al., 2020). Among these, *Torulaspora delbrueckii* has stood out for having a positive impact on the organoleptic properties of wine, producing high levels of fruit esters, thiols, and terpenes and lower amounts of higher alcohols, acetic acid, and acetaldehyde (Bely et al., 2008; Azzolini et al., 2015; Renault et al., 2015; Belda et al., 2017). Additionally, the use of *T. delbrueckii* favors the production of glycerol (Belda et al., 2015) and wines with lower alcohol content, which is desirable nowadays (Contreras et al., 2014). Regarding the ethanol tolerance of *T. delbrueckii*, Ciani and Maccarelli (1997) reported that *T. delbrueckii* is capable of fermenting and tolerating an ethanol concentration close to 9% (v/v); however, another study indicates that it only tolerates up to 7.4% (v/v) (Bely et al., 2008). In contrast, Canonico et al. (2018) reported that *T. delbrueckii* is capable of tolerating ethanol concentrations of 11.65% (v/v) present in base wine to obtain a sparkling wine with 12.5% (v/v) of ethanol, suggesting that the ethanol tolerance phenotype would be strain-dependent, limiting its use in industry. Limitations of this type in wine yeast strains have been addressed through the use of different strategies, such as random mutagenesis, hybridization, and metabolic engineering. Random mutagenesis is based on the application of mutagens in order to improve the natural mutation rate of microorganisms. However, it has limited efficacy in wine yeasts, since they are usually diploid and homothallic (De Vero et al., 2017), and phenotypic variants are produced at a slower rate. On the other hand, sexual hybridization strategies have been described as the most efficient way to generate artificial diversity in yeast. However, they can be difficult to apply given the homothalism characteristic and low sporulation rate of wine yeasts (McBryde et al., 2006). Metabolic engineering strategies, based on recombinant DNA technologies, have allowed to obtain strains of *S. cerevisiae* with an improved fermentative profile and the ability to increase sensory quality in wines (Schuller and Casal, 2005; Pizarro et al., 2007). However, currently there is a low acceptance of the use of genetically modified organisms (GMO) in wine and other agricultural products, which represents a major obstacle to the use of microorganisms obtained by metabolic engineering. Due to this, lately, other technologies are being used to generate strains with improved characteristics. One of these strategies is to improve other yeast species using microevolution or adaptive evolution, which is based on the growth of microorganisms under conditions of environmental stress or selective pressure, to obtain variants, presenting chromosomal rearrangements with a phenotype of interest (Voordeckers et al., 2015; Morschhäuser, 2016). Subsequently, adaptive evolution strategies represent an excellent alternative to generate strains with improved metabolic characteristics. It should be noted that the structural and/or metabolic changes in response to a specific stressor are a natural process and act as activators of the expression of genes involved in the synthesis of specific compounds that protect the organism, and can be observed in response to other stressors in the environment, for example, nutrient concentration, osmotic pressure, toxic compounds, and temperature variations (Saini et al., 2018).

In this way and considering that one of the main problems associated with *T. delbrueckii* is their tolerance to ethanol, which directly affects the contribution to the aromatic potential of wine, we used the adaptive evolution strategies to generate *T. delbrueckii* strains with enhanced ethanol-stress tolerance, and we evaluated aromatic contribution through co-inoculated fermentations.

## Materials and Methods

### Microorganisms

*Torulaspora delbrueckii* YCPUC10 was originally isolated from Cabernet Sauvignon must, and is part of the collection at the Laboratorio de Microbiología y Genética de Levaduras, Pontificia Universidad Católica de Chile. The yeast identity was confirmed by 26S D1/D2 sequencing (Kurtzman and Robnett, 2003). The strain was maintained on modified YPD broth (20 g/L glucose, 5 g/L peptone, and 5 g/L yeast extract) stored at −80°C with 40% glycerol.

Seven strains of *T. delbrueckii* (YCPUC10-A to YCPUC10-G) were obtained by adaptive evolution. The commercial strains *T. delbrueckii* Biodiva^TM^ and *S. cerevisiae* LALVIN EC1118^TM^ were provided by Lallemand Inc (Chile).

### Ethanol Resistance Phenotype Previous to Adaptive Evolution

The resistance of native yeast *T. delbrueckii* YCPUC10 (non-evolved) and commercial yeast *T. delbrueckii* Biodiva^TM^ to ethanol were determined by growing them in YPD medium (20 g/L glucose, 5 g/L peptone, and 5 g/L yeast extract) supplemented with 3, 6, 9, 10, 11, 12, and 14% (v/v) of ethanol. Cell growth was monitored by determining the optical density at 600 nm (OD_600_) using 1 h intervals. The experiments were done in triplicate in a 96-well microplate using 800 TSI plate reader coupled to the Gen5^TM^ software (BioTek, United States). The specific growth rate (*μ*max) was estimated from the slope of the growth curve during exponential phase according to the equation lnxt = x0 + mt, where: xt and x0 correspond to the biomass concentration or the optical density (OD) at time *t* (h) and *t* = 0, respectively (Barata et al., 2008). The *R*^2^ values of the curves were 0.996 or higher in all cases. *Lag* phase duration was determined mathematically according to Buchanan and Cygnarowicz (1990) as the time when the second derivative of the logarithm of the growth curve reaches a maximum value. Growth efficiency was defined as area under curve (AUC) and expressed as a percentage considering 100% the control condition (Godoy et al., 2016).

### Adaptive Evolution Experiment

**FIGURE 1:**
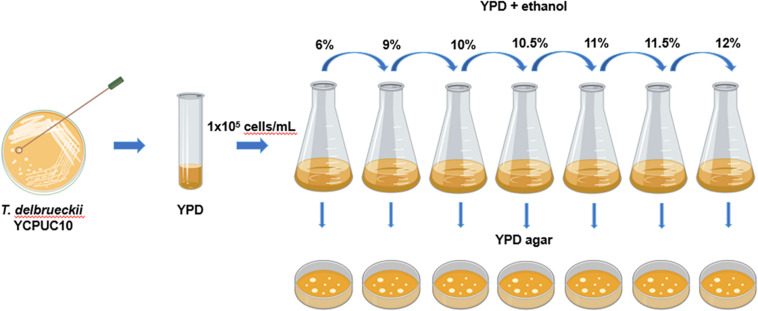
Diagram of the experimental procedures used to evolve *T. delbrueckii* YCPUC10 strain. Figure was created with BioRender.

*Torulaspora delbrueckii* YCPUC10 strain was adaptively evolved in YPD media supplemented with increasing concentrations of ethanol at 28°C for 114 days, through serial batch cultivation (Figure 1).

For this, at the start of the experiment, *T. delbrueckii* YCPUC10 cells from a single colony were inoculated in 5 mL YPD medium and incubated overnight at 28°C in an orbital shaker under 250 rpm. Then, 1 × 10^5^ cells/mL were subcultured into a new flask containing 50 mL fresh YDP medium supplemented with 6% (v/v) ethanol and incubated at 28°C in an orbital shaker under 250 rpm. When this population reached the mid-log phase, 1 × 10^5^ cells/mL were subcultured into a new flask containing 50 mL fresh YPD medium supplemented with 9% (v/v) ethanol and cultivated as described above. This process was repeated using 10, 10.5, 11, 11.5, and 12% (v/v) of ethanol. During the course of this adaptive evolution process, samples of the evolving populations were taken approximately every 50 generations and maintained in a glycerol stock (40 % glycerol) at −80°C for phenotypic analysis.

Additionally, each transfer was seeded on YPD agar (20 g/L glucose, 5 g/L peptone, 5 g/L yeast extract, and 20 g/L agar) to check viability. The species identity was identified by PCR amplification and sequencing of their 5.8-ITS rDNA regions, using ITS1 and ITS4 primers confirmed by sequencing (Macrogen, South Korea). The sequences of parental strain (YCPUC10) and evolved strain (YCPUC10-F) were deposited at GenBank under the accession numbers MW010022 and MW010023, respectively.

### Cell Growth Profiling

Growth curves were performed to characterize the fitness of the evolved clones of the initial *T. delbrueckii* YCPUC10 strain [6, 9, 10, 10.5, 11, 11.5, and 12% (v/v)]. Evolved clones were grown in YPD medium (20 g/L glucose, 5 g/L peptone, and 5 g/L yeast extract), and cell growth was monitored by determining the optical density at 600 nm (OD_600_) using 800 TSI plate reader coupled to the Gen5^TM^ software (BioTek, United States). The experiments were done in triplicate. The specific growth rate, *lag* phase duration and growth efficiency were determined as described above. From now on, we worked with one of the clones generated.

### Resistance to Potassium Metabisulfite

Resistance to potassium metabisulfite (PMB) of YCPUC10, YCPUC10-F and Biodiva^TM^ strains was evaluated. The strains were grown in synthetic must (140 g/L glucose, 140 g/L fructose, 1.7 g/L Yeast Nitrogen Base without ammonium sulfate and amino acid, 6 g/L citric acid, 6 g/L malic acid, 1.15 g/L ammonium chloride, 30 mg/L potassium disulfite, pH adjusted al 3.5 with KOH) (Novo et al., 2014) supplemented with different SO_2_ concentrations [0, 20, 25, 30, 35, 40, 45, and 50 (mg/L) free SO_2_]. Cell growth was monitored by determining the optical density at 600 nm (OD_600_). The experiments were done for triplicate in a 96-well microplate using 800 TSI plate reader coupled to the Gen5^TM^ software (BioTek, United States). The specific growth rate and *lag* phase duration were determined as described above.

### Fermentation Trials

Chardonnay grapes were used to prepare the must, and were obtained from a vineyard in Casablanca Valley, Valparaíso Region of Chile. Grapes were sprayed with 30 mg/L SO_2_ and refrigerated at 4°C. Then they were crushed and pressed to obtain the must. The parameters of the must obtained were pH 3.09, titratable acidity 4.63 g/L as tartaric acid, 213.6 g/L of reducing sugar. Fermentations were performed in triplicate in 50 mL conical tubes (Citotest, China) containing 45 mL of Chardonnay must. The tubes contained a 0.8 cm hole in their cap, through which a hose was connected to allow CO_2_ to escape. Three different assays were performed: (1) single inoculation with *S. cerevisiae* Lalvin EC1118^®^ (FSc); (2) Co-inoculation of *S. cerevisiae* Lalvin EC1118^®^ and *T. delbrueckii* YCPUC10 (FTd/Sc); (3) Co-inoculation of *S. cerevisiae* Lalvin EC1118^®^ and *T. delbrueckii* YCPUC10-F (FTdF/Sc). The inoculum ratio of *S. cerevisiae* and *T. delbrueckii* was 1:1, with an initial population of 1 × 10^5^ cell/mL of each one. All the tubes were incubated at 16°C under static conditions. The fermentation progress was monitored daily by measuring temperature, weight loss and density. Fermentations were considered finished when density was below 1.000 g/L. Reducing sugars, ethanol and volatile acidity determinations were performed according to the methods in the Compendium of International Methods of Analysis of Musts and Wines (OIV, 2014).

In order to quantify cell viability, *T. delbrueckii* yeast population counts were performed on the 1st, 3rd, 6th, and 16th days of fermentation. A sample was taken from each FTd/Sc and FTdF/Sc fermentations, and the cells were precipitated at 5,000 rpm. It was then washed with sterile distilled water and resuspended to make serial decimal dilutions. These were inoculated on Lysine agar plates (Oxoid, United Kingdom) and incubated for 5 days at 28°C.

Finally, the wines were filtered with a cellulose nitrate filter with a pore size of 0.2 μm (Sartorius Stedim Biotech, Germany) for aroma analysis.

### Analysis of Volatile Compounds

The volatile aroma compounds (i.e., a selection of acids, alcohols, aldehydes, C6 compounds, esters, ketones, norisoprenoids, phenolic derivatives, sulfur compound, and terpenes) were extracted by headspace solid-phase microextraction (HS-SPME) method with a 50/30 μm DVB/Carboxen/PDMS StableFlex fiber (Supelco, Bellefonte, PA, United States). 2 mL of sample, 4 mL of ultrapure water (30% NaCl) and 40 μL of 3.5 ppm 4-Nonanol (used as internal standard) were deposited in a 20 mL headspace vial. The sample was equilibrated for 10 min at 40°C, then it was extracted for 45 min (using SPME) at the same temperature, to later be injected into a gas chromatograph (GC) at a temperature of 250°C. The analyses were carried out on a GC 2010 plus Chromatograph (Shimadzu), coupled to a QP2010 ultra mass spectrometer (Shimadzu, Kyoto, Japan) and equipped with a Zebron^TM^ ZB-WAXplus^TM^ column (60 m × 0.25 mm × 0.25 μm) (Phenomenex, Torrance, CA, United States). The analysis was carried out in triplicate at Centro de Aromas y Sabores – DICTUC of Pontificia Universidad Católica de Chile. The odor activity values (OAV) were calculated as the ratio between the measured quantitative concentration of a substance in the wine and its odor threshold, when available.

### Sensory Analysis

An olfactory sensory analysis was performed with wine samples. The sensory panel consisted of 10 evaluators, both wine consumers and expert winemakers belonging to Departamento de Fruticultura y Enología of Pontificia Universidad Católica de Chile. The wines were presented to the evaluators at 15°C (15 mL) in standard sensory analysis chambers with separate booths in black wine glasses and were identified with three-digit random codes. The evaluators assigned scores from 0 (no character) to 10 (very strong character) for the following attributes: vegetable aromas, fresh fruit, tropical fruit, yeast, floral, butter, and spice. They also assigned values for the general acceptance of the wine from 0 (not accepted) to 10 (very accepted).

### Statistical Analysis

The statistical comparisons were carried out using analysis of variance (ANOVA) and the mean values of the experiments were compared using the LSD test. ANOVA for sensory descriptors was done for different treatments. The treatments were considered significant when the *p*-values ≤ 0.05. The analyses were done using Statgraphics Plus, version 5.1, (StatPoint Technologies, United States).

## Results

### Ethanol Resistance Phenotype

**FIGURE 2:**
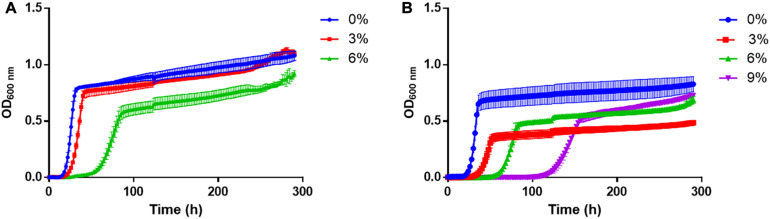
Growth curves of **(A)** *T. delbrueckii* Biodiva^TM^ and **(B)** YCPUC10 strains, exposed to different concentrations of ethanol.

As a first approximation, the growth of *T. delbrueckii* YCPUC10 and Biodiva^TM^ strains were evaluated in culture media supplemented with different concentrations of ethanol (Figure 2). The growth kinetics was affected in both strains as the concentration of ethanol in the culture medium increased.

For both strains, a tendency to a reduction of the growth rate was observed as the concentration of ethanol in the culture medium increased, in a similar way and without statistical differences between strains (Table 1). However, the commercial Biodiva^TM^ strain did not grow in the medium supplemented with 9% v/v ethanol, unlike the *T. delbrueckii* YCPUC10 strain, which was able to grow, but reached a growth rate 3.8 times lower than the medium not supplemented with ethanol.

**TABLE 1:** Kinetic parameters of the growth of strains of *T. delbrueckii* YCPUC10 (parental) and Biodiva^TM^ (commercial), exposed to different concentrations of ethanol.

**Ethanol (% v/v)**	**Specific growth rate μ_max_ (h^–1^)**		***Lag* phase (h)**		**Generation time Tg (h)**	
	**YCPUC10**	**Biodiva^TM^**	**YCPUC10**	**Biodiva^TM^**	**YCPUC10**	**Biodiva^TM^**
0	0.058 ± 0.007^a^	0.055 ± 0.008^a^	26.41 ± 0.00^a^	17.42 ± 1.64^b^	11.94 ± 1.57^a^	12.59 ± 1.95^a^
3	0.017 ± 0.005^a^	0.039 ± 0.008^a^	32.80 ± 5.33^a^	21.96 ± 5.06^a^	40.69 ± 11.96^a^	18.31 ± 4.16^a^
6	0.02 ± 0.002^a^	0.017 ± 0.002^a^	62.70 ± 1.23^a^	50.36 ± 1.84^b^	32.02 ± 4.88^a^	42.04 ± 5.89^a^
9	0.015 ± 0.003	NG	127.58 ± 5.33	NG	48.01 ± 9.89	NG

*Mean ± SD (*n* = 3). Different letters after means represent significant differences between strains at a given ethanol concentration (*p* ≤ 0.05). NG, no growth.*

In addition, the duration of the *lag* phase became longer, in the medium supplemented with 6% ethanol, compared to the non-supplemented medium, being up to 2.3 times longer for the *T. delbrueckii* YCPUC10 strain, and 2.8 times for the Biodiva^TM^ strain. Also, *T. delbrueckii* YCPUC10 grown in a medium supplemented with 9% ethanol, the *lag* phase was 4.8 times longer compared to the non-supplemented medium. The results indicate that the commercial Biodiva^TM^ strain had a shorter *lag* phase duration compared to the YCPUC10 strain. Likewise, the generation time parameter (Tg) increased for both strains as the concentration of ethanol in the culture medium is higher, with no statistically significant differences between them.

### Adaptive Evolution Experiment

**FIGURE 3:**
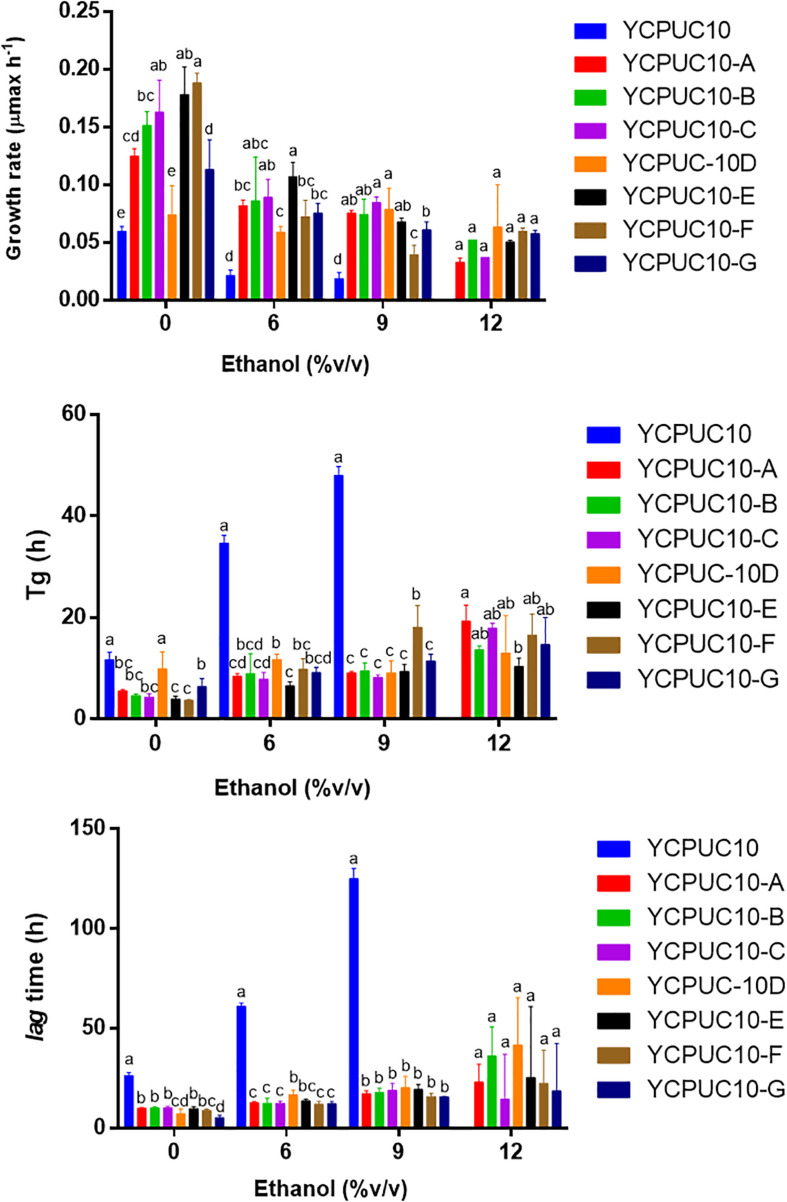
Growth kinetic parameters of *T. delbrueckii* YCPUC10 and evolved strains in YPD broth with different concentrations of ethanol. Mean ± SD (*n* = 3). Different letters above bars represent significant differences between clones at a given ethanol concentration (LSD *p* ≤ 0.05).

To generate yeast strains with enhanced ethanol tolerance, the native wine yeast *T. delbrueckii* YCPUC10 strain was subjected to adaptive evolution through serial batch cultivation. *T. delbrueckii* YCPUC10 was evolved over ∼ 300 generations, and we evaluated clones tolerance to ethanol (Figure 3).

Kinetics parameters analysis showed that all evolved clones had better tolerance to highest concentration of ethanol than the parent strain. The evolved strains were able to tolerate up to 12% ethanol, while the original strain only up to 9%. They also had improved kinetic parameters compared to the original strain, even when ethanol was not supplemented. The growth rate of all the evolved strains was higher than the original strain for all growth conditions. Likewise, the generation time (Tg) and the duration of the *lag* phase were shorter in the evolved strains than in the original strain. There were some differences among clones for some of the parameters, but they disappeared at the higher ethanol concentrations, except for generation time (Figure 3).

After adaptive evolution in ethanol, the clone YCPUC10-F was selected for its tolerance to high ethanol concentrations (11.5%) in batch cultures. This strain had statistically significant better kinetic parameters compared to the original strain (Table 2). At 9% ethanol, the duration of the lag phase was 7.8 times shorter in *T. delbrueckii* YCPUC10-F compared to the original strain, and a growth efficiency was 2.9 times higher. Also, the growth rate was 2.1 times higher in *T. delbrueckii* YCPUC10-F when compared to the original strain. The generation time parameter was also improved, decreasing 2.7 times compared to the original strain.

**TABLE 2:** Growth kinetic parameters of *T. delbrueckii* YCPUC10-F and the original strain in a medium with 9% ethanol.

**Strain**	**Growth efficiency (%)**	**Generation time Tg (h)**	**Specific growth rate μ_max_ (h^–1^)**	***Lag* phase (h)**
YCPUC10	19.56 ± 1.71^a^	48.81 ± 1.73^a^	0.019 ± 0.005^a^	125.0 ± 5.0^a^
YCPUC10-F	57.63 ± 1.51^b^	18.07 ± 4.30^b^	0.040 ± 0.008^b^	15.87 ± 1.60^b^

*Mean ± SD (*n* = 3). Different letters after means represent significant differences between strains for each parameter (LSD, *p* ≤ 0.05).*

### Potassium Metabisulfite Resistance

**FIGURE 4:**
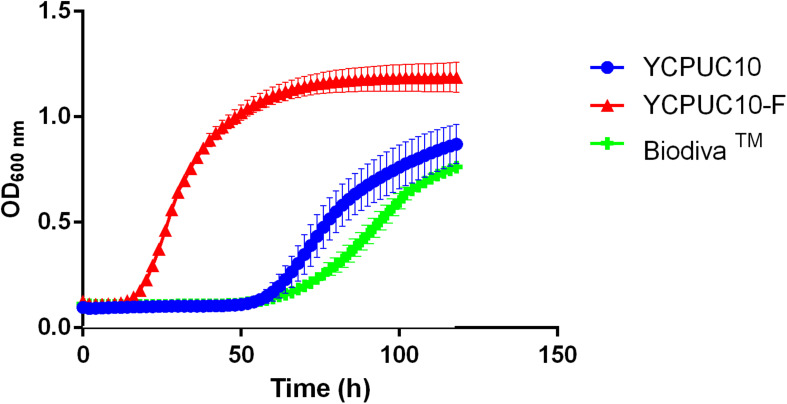
Growth curves of *T. delbrueckii* YCPUC10, YCPUC10-F and commercial strain Biodiva^TM^, in synthetic must with 50 mg/L of free SO_2_. Means ± SD (*n* = 3).

The effect of SO_2_ 50 mg/L on growth for YCPUC10, YCPUC10-F, and Biodiva^TM^ strains was evaluated in the synthetic must. Different behavior of the strains evaluated was found, with the evolved strain YCPUC10-F showing better fitness compared to the original and the commercial strains (Figure 4).

Table 3 shows that the evolved strain YCPUC10-F had 3.4 times shorter *lag* phase duration and a 1.9 times faster growth rate compared to the original strain YCPUC10, in synthetic must supplemented with 50 mg/L of free SO_2_. Also, it had a 3.5 times shorter lag phase and 2.7 times faster growth rate compared to the Biodiva^TM^. This behavior was also observed for the concentrations of 20 until 45 mg/L of free SO_2_ (data not shown).

**TABLE 3:** Growth kinetic parameters of the growth of *T. delbrueckii* strains in synthetic must with 50 mg/L of free SO_2_.

**Strain**	**Specific growth rate μ_max_ (h^–1^)**	***Lag* phase (h)**	**Generation time Tg (h)**
YCPUC10	0.019 ± 0.003^b^	62.56 ± 2.86^b^	36.81 ± 6.09^b^
YCPUC10-F	0.037 ± 0.001^a^	18.54 ± 0.51^a^	18.61 ± 0.65^a^
Biodiva^*TM*^	0.013 ± 0.004^b^	65.14 ± 3.59^b^	51.15 ± 0.45^c^

*Mean ± SD (*n* = 3). Different letters after means represent significant differences between strains for each parameter (LSD, *p* ≤ 0.05).*

### Fermentations

**FIGURE 5:**
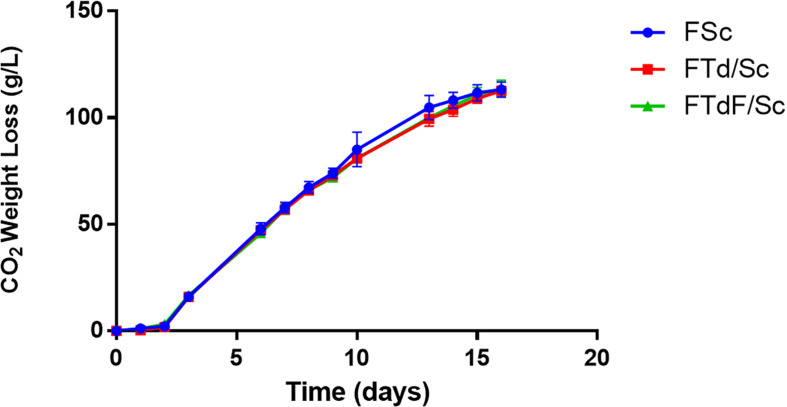
Fermentation kinetics of Chardonnay wines produced by pure *S. cerevisiae* fermentation (FSc) and co-inoculated fermentations FTd/Sc and FTdF/Sc.

Pure *S. cerevisiae* (FSc) and co-inoculated fermentation (FTd/Sc and FTdF/Sc) were inoculated with 1 × 10^5^ viable cells/mL for *T. delbrueckii* and 1 × 10^5^ viable cells/mL for *S. cerevisiae.* The fermentations were monitored through the loss of weight due to the production of CO_2_ (Figure 5).

From the fermentation curves of the microvinifications, it can be seen that all fermentations (FSc, FTd/Sc, and FTdF/Sc) ended after 16 days. After fermentation, 113.2 g/L of CO_2_ were released in FSc, 112.5 g/L in FTd/Sc and 113.6 g/L in FTdF/Sc, without significant differences between fermentations (Figure 5).

The volatile acidity remained below 0.31 g/L for the three fermentations, without statistically significant differences (Table 4). The residual sugar for fermentation was significantly higher for FTd/Sc (2.20 g/L) and FTd/Sc (2.60 g/L) compared to FSc (1.07 g/L). Regarding the ethanol concentration, the FTdF/Sc co-inoculated fermentation had a mean ethanol content of 10.8% and was significantly lower compared to the pure fermentation (FSc) that had an average content of 11.9%. For FTd/Sc fermentation, an intermediate value (11.2%) was observed (Table 4).

**TABLE 4:** Mean values and standard deviation of the composition of Chardonnay wines by pure *S. cerevisiae* fermentation (FSc) and co-inoculated fermentations FTd/Sc and FTdF/Sc.

**Wine**	**Volatile acidity (g/L)**	**Residual sugar (g/L)**	**Ethanol (% vol)**
FSc	0.31 ± 0.04^a^	1.07 ± 0.21^a^	11.9 ± 0.5^a^
FTd/Sc	0.24 ± 0.05^a^	2.60 ± 0.36^b^	11.2 ± 0.2^ab^
FTdF/Sc	0.25 ± 0.02^a^	2.20 ± 0.26^b^	10.8 ± 0.3^b^

*Different letters after means represent significant differences between yeasts treatments for each parameter (LSD, *p* ≤ 0.05).*

During fermentation in Chardonnay FTd/Sc and FTdF/Sc must, cell count of the *T. delbrueckii* YCPUC10 and YCPUC10-F strains was done (CFU/mL). These reached a maximum of 7.2 and 7.8 log (CFU/mL) respectively, after 6 days of fermentation and were able to maintain viability at the end of the fermentation of 58 and 56.7%, respectively, after 16 days (data not shown) without statistically significant differences.

### Metabolite Profile

The metabolite profile determination of the FSc, FTd/Sc, and FTdF/Sc wine samples was performed. It was possible to identify esters (25.51%), terpenes (19.4%), alcohols (17.35%), acids (11.22%), aldehydes (8.2%), phenolic derivatives (5.1%), ketones (4.1%), norisoprenoids (4.1%), C6 compounds (3.1%), sulfur compounds (2%), with a total of 98 different compounds (Supplementary Table 1).

The total concentration of acids was lower in FSc, while FTd/Sc and FTdF/Sc were similar. Four of them reached OAV ≥ 0.1 (Supplementary Table 1). FSc had significantly less total alcohols than FTdF/Sc, while FTd/Sc showed intermediate values. FTdF/Sc fermentation also had significantly higher concentration of 2-Ethylhexanol than FSc and FTd/Sc. The total concentration of aldehydes had significant differences among assays, however, in FSc the Mesitaldehyde concentration was significantly lower when compared to FTd/Sc and FTdF/Sc. C6 compounds and ketones did not show significant differences between assays. Similar results were observed for the contents of the total norisoprenoids.

Nineteen terpene compounds were quantifiable, but no significant differences were found among trials. However, Nerol oxide and Hotrienol concentrations were significantly higher in FTdF/Sc when compared to FSc and did not show significant differences with FTd/Sc assay. Linalool reached OAV ≥ 0.1 for all assays.

Total phenolics derivatives were significantly higher in co-inoculated fermentations FTd/Sc and FTdF/Sc than in FSc. Vinylguaiacol reached OAV ≥ 0.1 in all fermentations.

Sulfur compounds were lower in FSc, while FTd/Sc and FTdF/Sc no differences were found.

For total esters in the samples analyzed, the co-inoculated fermentations FTd/Sc and FTdF/Sc showed the highest total concentration in this family (Supplementary Table 1) and FSc showed statistically significant lower concentrations. In addition to highlighting for its OAV > 1 Isoamyl acetate. Also, six esters reached OAV ≥ 0.1 in all fermentations. Ethyl octanoate reached OAV ≥ 0.1 only in co-inoculated fermentations FTd/Sc and FTdF/Sc.

Finally, in the samples analyzed, the total compound concentration was highest in fermentation performed with evolved strain YCPUC10-F (FTdF/Sc). This trial was significantly different from FSc but did not show significant differences with FTd/Sc assay.

### Sensory Profiles

**FIGURE 6:**
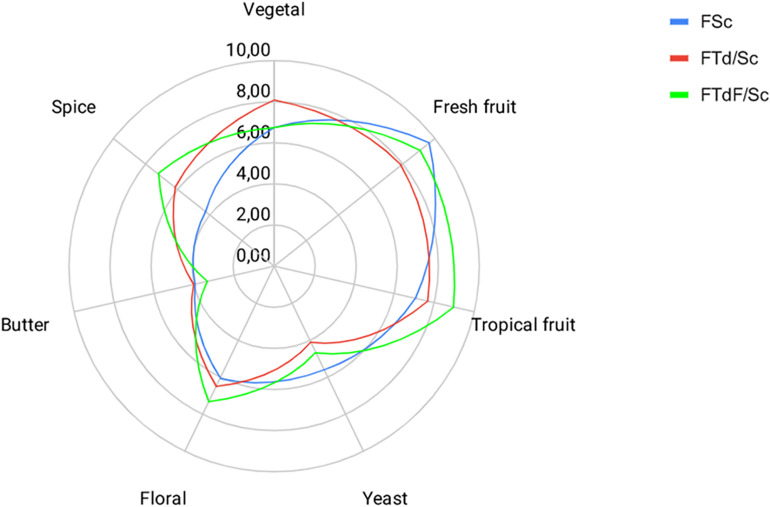
Sensory profiles for Chardonnay wine obtained by pure fermentation (FSc) and co-inoculated fermentation FTd/Sc and FTdF/Sc.

Sensory profiles obtained in FTd/Sc and FTdF/Sc indicate an intensification of some descriptors, with respect to the control FSc, despite no significant differences between the trials (Figure 6).

Aromas such as fresh and tropical fruits had a higher intensity rating. FTdF/Sc had a rating of 8.94 in tropical fruits aromas, followed by FTd/Sc with 7.71 and FSc with 7.04. The floral aroma intensity was also higher in FTdF/Sc with a score of 7.28, then FTd/Sc with 6.51 and FSc with 6.05. A similar trend is observed for the spice descriptor, whose highest score was obtained by FTdF/Sc with 7.20, followed by FTd/Sc with 6.20 and FSc with 4.29.

FTd/Sc had a higher rating for vegetable flavor with 8.07, followed by FTdF/Sc with 6.78 and FSc with 6.76. At the same time, FSc had a higher score for the yeast descriptor, with a score of 5.56, followed by FTdF/Sc with 4.66 and FTd/Sc with 4.12. Butter descriptor had a low score in all the wines, FTd/Sc was scored with 4.00, followed by FSc with 3.96 and FTdF/Sc with 3.31.

Regarding the general acceptance of the wine, FTdF/Sc had the highest acceptance with a rating of 9.92, followed by FTd/Sc with 8.74 and FSc with 7.94.

## Discussion

We first characterized the behavior of the native strain of *T. delbrueckii* YCPUC10 and the commercial strain Biodiva^TM^ in media supplemented with different concentrations of ethanol. The results indicated both strains performed worse as the concentration of ethanol in the culture medium increased. The growth rate was affected, decreasing further as the concentration of ethanol increased. The negative effects of increasing ethanol have been reported for other yeasts, such as *S. cerevisiae* (Fleet, 1990; Gil et al., 1996; Kubota et al., 2004). Ethanol represents a form of chemical stress for the microorganisms present in the must, inhibiting yeast growth, negatively affecting cell division, decreasing the cell volume and the specific growth rate (Stanley et al., 2010a).

On the other hand, the native YCPUC10 strain was able to grow in a medium supplemented up to 9% ethanol, unlike the commercial Biodiva^TM^ strain, which only grew in the medium with up to 6% ethanol. This variability is in agreement with that described by other works (Ciani and Maccarelli, 1997; Bely et al., 2008; Canonico et al., 2018), who reported that *T. delbrueckii* is capable of fermenting and tolerating an ethanol concentration from 7.4 to 12.5% (v/v), being a strain-dependent property. However, Belda et al. (2015, 2017) observed that *T. delbrueckii* strains significantly decrease their cell viability when ethanol levels exceed 8% (v/v).

### Adaptive Evolution

One of the main problems associated with non-*Saccharomyces* yeasts, in particular *T. delbrueckii*, is their low tolerance to ethanol, which directly affects their contribution to the aromatic potential of wine. We used adaptive evolution to generate *T. delbrueckii* strains with improved ethanol-stress tolerance.

Our results showed that all the evolved yeast had better resistance to high concentrations of ethanol than the parental strain. Also, the results showed that after about 300 generations of adaptive evolution, the yeast growth efficacy increased, indicating that adaptive mutations had begun to accumulate.

The variability in the data observed in the medium with 12% ethanol is attributable to the greater stress generated by this high concentration of ethanol to which the yeast was exposed. Studies conducted by Novo et al. (2014), performing adaptive evolution experiments in *S. cerevisiae*, reported a similar behavior in the growth curves. They observed that by increasing the percentage of ethanol in the medium from 6 to 8%, the kinetic behavior presents high variability.

While there are no studies that report the generation of *T. delbrueckii* strains resistant to ethanol by adaptive evolution, there are some studies that have reported the use this strategy to improve ethanol resistance in *S. cerevisiae* (Brown and Oliver, 1982; Dinh et al., 2008; Stanley et al., 2010b; Fiedurek et al., 2011; Chen and Xu, 2014) and *Kluyveromyces marxianus* (Mo et al., 2019; da Silveira et al., 2020). In this sense, da Silveira et al. (2020) reported the generation of four ethanol tolerant strains of *K. marxianus* CCT 7735 by adaptive laboratory evolution. One of them, ETS4 strain, showed a higher specific growth rate than the parental strain under stress of ethanol. For their part, Chen and Xu (2014) reported the generation of ethanol-tolerant *S. cerevisiae* mutant than exhibited increased tolerance to ethanol, in addition to higher osmotic and temperature tolerances than the parent strain. Mo et al. (2019) developed *K. marxianus* strains with a high tolerance to ethanol of 7–10% (v/v), which in fact led to increased production of ethanol in a multiple stress environment.

Yeast ethanol resistance is a complex phenotype regulated by multiple genes. In *S. cerevisiae*, apart from genes directly related to the metabolism of ethanol, it has been described that affects genes related to the glycolytic pathway, lipid metabolism, cell wall biogenesis and composition of the plasma membrane, protein folding, among others (Ding et al., 2009; Ma and Liu, 2010; Stanley et al., 2010a; Snoek et al., 2016). Likewise, glutathione (GSH) has been reported to be involved in numerous stress response mechanisms (Ghosh et al., 1999; Carmel-Harel and Storz, 2000), including ethanol stress, where it plays an important role in the maintenance of basic functions such as protection of cell membranes and in the maintenance of redox balance (Saharan et al., 2010).

In particular, an increase in the content of unsaturated fatty acids has been reported in ethanol-tolerant *S. cerevisiae* strains, as well as a higher percentage of oleic acid and ergosterol to maintain optimal membrane thickness (Saini et al., 2018).

We can hypothesize that similar changes occurred in *T. delbrueckii*, due to the genetic closeness that exists between *S. cerevisiae* and *T. delbrueckii* (James et al., 1996; Belloch et al., 2000), however, this must be investigated.

Apart from ethanol tolerance, yeast strains for industrial use should maintain other important fermentation traits. For yeasts of oenological interest, resistance to potassium metabisulfite represents a critical trait to consider. Potassium metabisulfite is an antimicrobial widely used in the wine industry and inhibits the growth of spoilage yeasts (Sun et al., 2016; Edwards and Oswald, 2018). However, the addition of SO_2_ can positively or negatively affect the growth of various desired yeast species during fermentation. It has been reported that SO_2_ addition between 40 and 80 mg/L negatively affects growth of *T. delbrueckii* (Albertin et al., 2014; Chandra et al., 2015; Ferreira et al., 2017). We evaluated the growth of YCPUC10, Biodiva^TM^ and evolved strain YCPUC10-F, selected for its markedly improved tolerance to ethanol, in the presence of SO_2_. Interestingly, the evolved yeast strains also showed increased tolerance to SO_2_. The ability of a stress condition to provide protection against other stresses is known as cross-protection. This phenomenon refers to the fact that multiple stresses can share some common pathways against stress. Various studies have shown that adaptation to ethanol and acetic acid stress confers resistance to a wide range of stress conditions, including thermal, osmotic, and oxidative stress (Gurdo et al., 2018; Mo et al., 2019). This could explain what was observed for the YCPUC10-F strain.

Considering the results obtained, an evolved yeast strain (YCPUC10-F) was selected to test fermentation parameters by co-inoculated fermentation in Chardonnay must.

### Fermentation Behavior of Co-inoculated Fermentations

The duration of FTd/Sc and FTdF/Sc co-inoculated fermentations was similar to that in other studies (Azzolini et al., 2015; Belda et al., 2015; van Breda et al., 2018).

The low levels of volatile acidity observed in our study are in agreement with what was reported earlier (Ciani et al., 2006; Bely et al., 2008). Acetic acid production for FTd/Sc and FTdF/Sc averaged 0.25 g/L, and fermentation with *S. cerevisiae* only produced 0.31 g/L. Similar results have been reported by Bely et al. (2008) and Loira et al. (2014).

Even though the three fermentations ended on the same day, the alcohol levels produced in the pure fermentation (FSc) were higher than those observed in the simultaneous FTd/Sc and FTdF/Sc fermentations (Table 4). Similar results were reported in other studies (Taillandier et al., 2014; Belda et al., 2015), who observed levels of 10.1–10.7% w/v of ethanol for simultaneous and mixed fermentations. Our results confirm the potential of mixed fermentations (Sc/Td) to reduce ethanol. Also, in this trial, *S. cerevisiae* fermentation produces a higher level of ethanol, which agrees with its high fermentative purity, and shown a significant difference with FTdF/Sc fermentation.

### Metabolite Profile and Sensory Analysis

Currently, a strategy used to maximize the oenological potential of non-*Saccharomyces* yeasts is to use mixed cultures. In this study, the effect of original strain YCPUC10 and evolved strains YCPUC10-F of *T. delbrueckii* with *S. cerevisiae* EC1118 strain on the Chardonnay wine aroma quality was investigated.

FTd/Sc and FTdF/Sc fermentations showed different aromatic compound profiles than FSc (pure fermentation). Higher alcohols correspond to the most important group of volatile compounds produced by yeast during the fermentation of sugars (Ugliano and Henschke, 2009). The contribution of these compounds to wine aroma was important in FTd/Sc and FTdF/Sc. Our results are consistent with those reported in literature, where the use of mixed cultures of *T. delbrueckii* and *S. cerevisiae* are associated with higher alcohol formation, particularly phenylethyl alcohol (Comitini et al., 2011; Azzolini et al., 2012; Sadoudi et al., 2012; Fresno et al., 2017). Also, a higher concentration of 2-Ethylhexanol (rose, citrus), which is consistent with the high aroma perception observed for FTdF/Sc. This suggests that co-inoculated fermentation with an evolved strain (FTdF/Sc) improves the aromatic profile of Chardonnay wine compared to the original strain (FTd/Sc) and pure fermentation (FSc).

Esters production comes from alcoholic fermentation and they are critical regarding their contribution to aromas since they are responsible for the fruitiness. Additionally, it is suggested that they are involved in the aromatic evolution of wine over time (Lambrechts and Pretorius, 2019). For total esters, the co-inoculated fermentations FTd/Sc and FTdF/Sc showed the highest total concentration of these compounds compared with pure fermentation FSc. In particular, ethyl acetate and phenethyl acetate concentrations are significantly higher in FTd/Sc and FTdF/Sc. Our results are in accordance with what was reported by Loira et al. (2014), Renault et al. (2015), and Arslan et al. (2018), where the mixed cultures between *T. delbrueckii* and *S. cerevisiae* present higher concentrations of these compounds compared to monocultures.

Our data also showed that *β*-Damascenone (roses, honey, apple, OVA > 1) stands out from the norisoprenoid compounds. This compound is considered as a powerful odor and enhancer of fruit aromas (Escudero et al., 2007). Concerning terpenes concentration, were significantly higher in FTdF/Sc and FTd/Sc, when compared to FSc. Similar results have been reported by several authors (King and Richard Dickinson, 2000; Hernandez-Orte et al., 2008; Comitini et al., 2011; Azzolini et al., 2012). It has been described that the use of *T. delbrueckii* in winemaking has advantages, including obtaining wines with a higher concentration of mannoproteins (Azzolini et al., 2015; Belda et al., 2015). Some aromatic compounds can interact with polysaccharides or proteins, and there is evidence that mannoproteins can affect the aromatic composition of wine, increasing the concentration of positive aromatic compounds, such as terpenes and norisoprenoids (Juega et al., 2012). In addition, it has been reported that mannoproteins could help reduce the volatility of aromatic compounds by more than 80% (Chalier et al., 2007).

In all wine samples, two of these compounds were highlighted by their OAV > 1, isoamyl acetate and *β*-Damascenone. The sensory panel perceived intensification of the “floral” and “fruity” sensory descriptors in co-inoculated fermentation with the evolved strain YCPUC10-F (FTdF/Sc), compared to FTd/Sc and FSc. In this regard, several studies have indicated that the use of *T. delbrueckii* in mixed fermentations is beneficial from the aromatic point of view (Loira et al., 2014; Minnaar et al., 2015; Belda et al., 2017), reporting better quality of aroma, intensity, and fruity character.

Resistance to ethanol and the ability to produce aromas are of great commercial interest, especially in non-conventional yeasts, where their metabolism contributes positively by granting identity and aromatic complexity to wines, enhancing their quality.

In this way, one of the main problems associated with non-*Saccharomyces* yeasts, in particular *T. delbrueckii*, is their low tolerance to ethanol, which directly affects the contribution to the aromatic potential of wine. Our results indicate that the YCPUC10-F strain, obtained by adaptive evolution, has improved kinetic parameters in a medium with 9% ethanol compared to the parental strain. Furthermore, the evolved strain shows an increase in resistance to potassium metabisulfite, which gives it a competitive advantage in the early stages of fermentation. According to our knowledge, there are no reports of the use of this strategy to improve the phenotype of resistance to ethanol in the yeast *T. delbrueckii*, this being the first report.

Currently, our group is working on the analysis of the genome of the evolved strain in order to identify changes at the genetic level that explain the improvement of the phenotype. Future studies include increasing the initial population of the evolved strain and vinification on a larger scale to assess its behavior and adaptability of the YCPUC10-F strain.

## Data Availability Statement

The raw data supporting the conclusions of this article will be made available by the authors, without undue reservation.

## Author Contributions

LG and AA-F designed the experiments. AA-F and DC conducted the experiments. LG, AA-F, and DC analyzed the experimental data, wrote the manuscript, and reviewed the manuscript.

## Conflict of Interest

The authors declare that the research was conducted in the absence of any commercial or financial relationships that could be construed as a potential conflict of interest.

## Supplementary Material

Supplementary Table 1 Volatile composition (mg/L) of the final wines after alcoholic fermentation. FSc, *S. cerevisiae* EC1118 pure fermentation; FTd/Sc, co-inoculated fermentation of EC1118 and YCPUC10; FTdF/Sc, co-inoculated fermentation of EC1118 and YCPUC10-F. Mean ± SD (*n* = 3). Different letters after means represent significant differences among yeast combinations for fermentation in each parameter (LSD, *p* ≤ 0.05). Aroma compounds that OAV exceeding one are highlighted and those compounds that OAV exceeding 0.1 but <1 are underlined.

## References

[B1] Albertin W. Miot-Sertier C. Bely M. Marullo P. Coulon J. Moine V. (2014). Oenological prefermentation practices strongly impact yeast population dynamics and alcoholic fermentation kinetics in Chardonnay grape must. *Int. J. Food Microbiol.* 178 87–97. 10.1016/j.ijfoodmicro.2014.03.009 24681710

[B2] Arslan E. Çelik Z. D. Cabaroğlu T. (2018). Effects of Pure and mixed autochthonous *Torulaspora delbrueckii* and *Saccharomyces cerevisiae* on fermentation and volatile compounds of Narince wines. *Foods* 7:147. 10.3390/foods7090147 30189601 PMC6163554

[B3] Azzolini M. Fedrizzi B. Tosi E. Finato F. Vagnoli P. Scrinzi C. (2012). Effects of *Torulaspora delbrueckii* and *Saccharomyces cerevisiae* mixed cultures on fermentation and aroma of Amarone wine. *Eur. Food Res. Technol.* 235 303–313. 10.1007/s00217-012-1762-3

[B4] Azzolini M. Tosi E. Lorenzini M. Finato F. Zapparoli G. (2015). Contribution to the aroma of white wines by controlled *Torulaspora delbrueckii* cultures in association with *Saccharomyces cerevisiae*. *World J. Microbiol. Biotechnol.* 31 277–293. 10.1007/s11274-014-1774-1 25388474

[B5] Barata A. Pagliara D. Piccininno T. Tarantino F. Ciardulli W. Malfeito-Ferreira M. (2008). The effect of sugar concentration and temperature on growth and volatile phenol production by *Dekkera bruxellensisin* wine. *FEMS Yeast Res.* 8 1097–1102. 10.1111/j.1567-1364.2008.00415.x 18637043

[B6] Belda I. Navascués E. Marquina D. Santos A. Calderon F. Benito S. (2015). Dynamic analysis of physiological properties of *Torulaspora delbrueckii* in wine fermentations and its incidence on wine quality. *Appl. Microbiol. Biotechnol.* 99 1911–1922. 10.1007/s00253-014-6197-2 25408314

[B7] Belda I. Ruiz J. Beisert B. Navascués E. Marquina D. Calderón F. (2017). Influence of *Torulaspora delbrueckii* in varietal thiol (3-SH and 4-MSP) release in wine sequential fermentations. *Int. J. Food Microbiol.* 257 183–191. 10.1016/j.ijfoodmicro.2017.06.028 28668728

[B8] Belloch C. Querol A. García M. D. Barrio E. (2000). Phylogeny of the genus *Kluyveromyces* inferred from the mitochondrial cytochrome-c oxidase II gene. *Int. J. Syst. Evol. Microbiol.* 50(Pt 1), 405–416. 10.1099/00207713-50-1-405 10826829

[B9] Bely M. Stoeckle P. Masneuf-Pomarède I. Dubourdieu D. (2008). Impact of mixed *Torulaspora delbrueckii*-*Saccharomyces cerevisiae* culture on high-sugar fermentation. *Int. J. Food Microbiol.* 122 312–320. 10.1016/j.ijfoodmicro.2007.12.023 18262301

[B10] Brown S. W. Oliver S. G. (1982). Isolation of ethanol-tolerant mutants of yeast by continuous selection. *Eur. J. Appl. Microbiol. Biotechnol.* 16 119–122. 10.1007/BF00500738

[B11] Buchanan R. L. Cygnarowicz M. L. (1990). A mathematical approach toward defining and calculating the duration of the lag phase. *Food Microbiol.* 7 237–240. 10.1016/0740-0020(90)90029-H

[B12] Canonico L. Comitini F. Ciani M. (2018). *Torulaspora delbrueckii* for secondary fermentation in sparkling wine production. *Food Microbiol.* 74 100–106. 10.1016/j.fm.2018.03.009 29706323

[B13] Carmel-Harel O. Storz G. (2000). Roles of the glutathione- and thioredoxin-dependent reduction systems in the *Escherichia coli* and *saccharomyces cerevisiae* responses to oxidative stress. *Annu. Rev. Microbiol.* 54 439–461. 10.1146/annurev.micro.54.1.439 11018134

[B14] Chalier P. Angot B. Delteil D. Doco T. Gunata Z. (2007). Interactions between aroma compounds and whole mannoprotein isolated from *Saccharomyces cerevisiae* strains. *Food Chem.* 100 22–30. 10.1016/j.foodchem.2005.09.004

[B15] Chandra M. Oro I. Ferreira-Dias S. Malfeito-Ferreira M. (2015). Effect of ethanol, sulfur dioxide and glucose on the growth of wine spoilage yeasts using response surface methodology. *PLoS One* 10:e0128702. 10.1371/journal.pone.0128702 26107389 PMC4479441

[B16] Chen S. Xu Y. (2014). Adaptive evolution of *Saccharomyces cerevisiae* with enhanced ethanol tolerance for Chinese rice wine fermentation. *Appl. Biochem. Biotechnol.* 173 1940–1954. 10.1007/s12010-014-0978-z 24879599

[B17] Ciani M. Beco L. Comitini F. (2006). Fermentation behaviour and metabolic interactions of multistarter wine yeast fermentations. *Int. J. Food Microbiol.* 108 239–245. 10.1016/j.ijfoodmicro.2005.11.012 16487611

[B18] Ciani M. Comitini F. Mannazzu I. Domizio P. (2010). Controlled mixed culture fermentation: a new perspective on the use of non-*Saccharomyces* yeasts in winemaking. *FEMS Yeast Res.* 10 123–133. 10.1111/j.1567-1364.2009.00579.x 19807789

[B19] Ciani M. Maccarelli F. (1997). Oenological properties of non-*Saccharomyces* yeasts associated with wine-making. *World J. Microbiol. Biotechnol.* 14 199–203. 10.1023/A:1008825928354

[B20] Comitini F. Gobbi M. Domizio P. Romani C. Lencioni L. Mannazzu I. (2011). Selected non-*Saccharomyces* wine yeasts in controlled multistarter fermentations with *Saccharomyces cerevisiae*. *Food Microbiol.* 28 873–882. 10.1016/j.fm.2010.12.001 21569929

[B21] Contreras A. Hidalgo C. Henschke P. A. Chambers P. J. Curtin C. Varela C. (2014). Evaluation of non-*Saccharomyces* yeasts for the reduction of alcohol content in wine. *Appl. Environ. Microbiol.* 80 1670–1678. 10.1128/AEM.03780-13 24375129 PMC3957604

[B22] da Silveira F. A. de Oliveira Soares D. L. Bang K. W. Balbino T. R. de Moura Ferreira M. A. Diniz R. H. S. (2020). Assessment of ethanol tolerance of *Kluyveromyces marxianus* CCT 7735 selected by adaptive laboratory evolution. *Appl. Microbiol. Biotechnol.* 104 7483–7749. 10.1007/s00253-020-10768-9 32676708

[B23] De Vero L. Bonciani T. Verspohl A. Mezzetti F. Giudici P. (2017). High-glutathione producing yeasts obtained by genetic improvement strategies: a focus on adaptive evolution approaches for novel wine strains. *AIMS Microbiol.* 3 155–170. 10.3934/microbiol.2017.2.155 31294155 PMC6605010

[B24] Ding J. Huang X. Zhang L. Zhao N. Yang D. Zhang K. (2009). Tolerance and stress response to ethanol in the yeast *Saccharomyces cerevisiae*. *Appl. Microbiol. Biotechnol.* 85 253–263. 10.1007/s00253-009-2223-1 19756577

[B25] Dinh T. N. Nagahisa K. Hirasawa T. Furusawa C. Shimizu H. (2008). Adaptation of *Saccharomyces cerevisiae* cells to high ethanol concentration and changes in fatty acid composition of membrane and cell size. *PLoS One* 3:e2623. 10.1371/journal.pone.0002623 18612424 PMC2440543

[B26] Edwards C. G. Oswald T. A. (2018). Interactive effects between total SO2, ethanol and storage temperature against *Brettanomyces bruxellensis*. *Lett. Appl. Microbiol.* 66 71–76. 10.1111/lam.12816 29080348

[B27] Escudero A. Campo E. Fariña L. Cacho J. Ferreira V. (2007). Analytical characterization of the aroma of five premium red wines. insights into the role of odor families and the concept of fruitiness of wines. *J. Agric. Food Chem.* 55 4501–4510. 10.1021/jf0636418 17488088

[B28] Ferreira D. Galeote V. Sanchez I. Legras J.-L. Ortiz-Julien A. Dequin S. (2017). Yeast multistress resistance and lag-phase characterisation during wine fermentation. *FEMS Yeast Res.* 17:fox051. 10.1093/femsyr/fox051 28817926

[B29] Fiedurek J. Skowronek M. Gromada A. (2011). Selection and Adaptation of *Saccharomyces* cerevisae to increased ethanol tolerance and production. *Pol. J. Microbiol.* 60 51–58. 10.33073/pjm-2011-007 21630574

[B30] Fleet G. H. (1990). Growth of yeasts during wine fermentations. *J. Wine Res.* 1 211–223. 10.1080/09571269008717877

[B31] Fleet G. H. Lafon-Lafourcade S. Ribéreau-Gayon P. (1984). Evolution of yeasts and lactic Acid bacteria during fermentation and storage of bordeaux wines. *Appl. Environ. Microbiol.* 48 1034–1038. 10.1128/AEM.48.5.1034-1038.1984 16346661 PMC241671

[B32] Fresno J. M. D. Del Fresno J. M. Morata A. Loira I. Bañuelos M. A. Escott C. (2017). Use of non-*Saccharomyces* in single-culture, mixed and sequential fermentation to improve red wine quality. *Eur. Food Res. Technol.* 243 2175–2185. 10.1007/s00217-017-2920-4

[B33] Ghosh M. Shen J. Rosen B. P. (1999). Pathways of As(III) detoxification in *Saccharomyces cerevisiae*. *Proc. Natl. Acad. Sci. U.S.A.* 96 5001–5006. 10.1073/pnas.96.9.5001 10220408 PMC21806

[B34] Gil J. V. Mateo J. J. Jiménez M. Pastor A. Huerta T. (1996). Aroma compounds in wine as influenced by Apiculate yeasts. *J. Food Sci.* 61 1247–1250. 10.1111/j.1365-2621.1996.tb10971.x

[B35] Godoy L. Acuña-Fontecilla A. Catrileo D. (2020). “Formation of aromatic and flavor compounds in wine: a perspective of positive and negative contributions of Non-*Saccharomyces* yeasts,” in *Winemaking – Stabilization, Aging Chemistry and Biochemistry [Working Title]*, eds F. Cosme, F. M. Nunes, and L. Filipe-Ribeiro (London: IntechOpen). 10.5772/intechopen.92562

[B36] Godoy L. Vera-Wolf P. Martinez C. Ugalde J. A. Ganga M. A. (2016). Comparative transcriptome assembly and genome-guided profiling for *Brettanomyces bruxellensis* LAMAP2480 during p-coumaric acid stress. *Sci. Rep.* 6:34304. 10.1038/srep34304 27678167 PMC5039629

[B37] González-Royo E. Pascual O. Kontoudakis N. Esteruelas M. Esteve-Zarzoso B. Mas A. (2015). Oenological consequences of sequential inoculation with non-*Saccharomyces* yeasts (*Torulaspora delbrueckii* or *Metschnikowia pulcherrima*) and *Saccharomyces cerevisiae* in base wine for sparkling wine production. *Eur. Food Res. Technol.* 240 999–1012. 10.1007/s00217-014-2404-8

[B38] Gurdo N. Novelli Poisson G. F. Juárez ÁB. de Molina M. C. R. Galvagno M. A. (2018). Improved robustness of an ethanologenic yeast strain through adaptive evolution in acetic acid is associated with its enzymatic antioxidant ability. *J. Appl. Microbiol.* 125 766–776. 10.1111/jam.13917 29770550

[B39] Hernandez-Orte P. Cersosimo M. Loscos N. Cacho J. Garciamoruno E. Ferreira V. (2008). The development of varietal aroma from non-floral grapes by yeasts of different genera. *Food Chem.* 107 1064–1077. 10.1016/j.foodchem.2007.09.032

[B40] James S. A. Collins M. D. Roberts I. N. (1996). Use of an rRNA internal transcribed spacer region to distinguish phylogenetically closely related species of the genera *Zygosaccharomyces* and *Torulaspora*. *Int. J. Syst. Bacteriol.* 46 189–194. 10.1099/00207713-46-1-189 8573494

[B41] Jolly N. P. Augustyn O. P. H. Pretorius I. S. (2006). The role and use of Non-*Saccharomyces* yeasts in wine production. *S. Afr. J. Enol. Vitic.* 27 15–38. 10.21548/27-1-1475 16203244

[B42] Jolly N. P. Varela C. Pretorius I. S. (2013). Not your ordinary yeast: non-*Saccharomyces* yeasts in wine production uncovered. *FEMS Yeast Res.* 14 215–237. 10.1111/1567-1364.12111 24164726

[B43] Juega M. Nunez Y. P. Carrascosa A. V. Martinez-Rodriguez A. J. (2012). Influence of yeast mannoproteins in the aroma improvement of white wines. *J. Food Sci.* 77 M499–M504. 10.1111/j.1750-3841.2012.02815.x 22860598

[B44] King A. Richard Dickinson J. (2000). Biotransformation of monoterpene alcohols by *Saccharomyces cerevisiae*, *Torulaspora delbrueckii* and *Kluyveromyces lactis*. *Yeast* 16 499–506. 10.1002/(SICI)1097-0061(200004)16:6<499::AID-YEA548>3.0.CO;2-E 10790686

[B45] Kubota S. Takeo I. Kume K. Kanai M. Shitamukai A. Mizunuma M. (2004). Effect of ethanol on cell growth of budding yeast: genes that are important for cell growth in the presence of ethanol. *Biosci. Biotechnol. Biochem.* 68 968–972. 10.1271/bbb.68.968 15118337

[B46] Kurtzman C. P. Robnett C. J. (2003). Phylogenetic relationships among yeasts of the “*Saccharomyces* complex” determined from multigene sequence analyses. *FEMS Yeast Res.* 3 417–432. 10.1016/S1567-1356(03)00012-6 12748053

[B47] Lambrechts M. G. Pretorius I. S. (2019). Yeast and its importance to wine aroma – a review. *S. Afric. J. Enol. Vitic.* 21:3560 10.21548/21-1-3560

[B48] Loira I. Vejarano R. Bañuelos M. A. Morata A. Tesfaye W. Uthurry C. (2014). Influence of sequential fermentation with *Torulaspora delbrueckii* and *Saccharomyces cerevisiae* on wine quality. *LWT Food Sci. Technol.* 59 915–922. 10.1016/j.lwt.2014.06.019

[B49] Ma M. Liu Z. L. (2010). Mechanisms of ethanol tolerance in *Saccharomyces cerevisiae*. *Appl. Microbiol. Biotechnol.* 87 829–845. 10.1007/s00253-010-2594-3 20464391

[B50] McBryde C. Gardner J. M. de Barros Lopes M. Jiranek V. (2006). Generation of novel wine yeast strains by adaptive evolution. *Am. J. Enol. Vitic.* 57 423–430.

[B51] Minnaar P. P. Ntushelo N. Ngqumba Z. van Breda V. Jolly N. P. (2015). Effect of *Torulaspora delbrueckii* yeast on the anthocyanin and flavanol concentrations of cabernet franc and Pinotage Wines. *S. Afric. J. Enol. Vitic.* 36 50–58. 10.21548/36-1-936

[B52] Mo W. Wang M. Zhan R. Yu Y. He Y. Lu H. (2019). *Kluyveromyces marxianus* developing ethanol tolerance during adaptive evolution with significant improvements of multiple pathways. *Biotechnol. Biofuels* 12:63. 10.1186/s13068-019-1393-z 30949239 PMC6429784

[B53] Morschhäuser J. (2016). The development of fluconazole resistance in *Candida albicans* – an example of microevolution of a fungal pathogen. *J. Microbiol.* 54 192–201. 10.1007/s12275-016-5628-4 26920879

[B54] Novo M. Gonzalez R. Bertran E. Martínez M. Yuste M. Morales P. (2014). Improved fermentation kinetics by wine yeast strains evolved under ethanol stress. *LWT Food Sci. Technol.* 58 166–172. 10.1016/j.lwt.2014.03.004

[B55] OIV (2014). *Compendium of International Methods of Wine and Must Analysis; International Organization of Vine and Wine.* Paris: OIV.

[B56] Pizarro F. Vargas F. A. Agosin E. (2007). A systems biology perspective of wine fermentations. *Yeast* 24 977–991. 10.1002/yea.1545 17899563

[B57] Renault P. Coulon J. de Revel G. Barbe J.-C. Bely M. (2015). Increase of fruity aroma during mixed *T. delbrueckii*/*S. cerevisiae* wine fermentation is linked to specific esters enhancement. *Int. J. Food Microbiol.* 207 40–48. 10.1016/j.ijfoodmicro.2015.04.037 26001522

[B58] Sadoudi M. Tourdot-Maréchal R. Rousseaux S. Steyer D. Gallardo-Chacón J.-J. Ballester J. (2012). Yeast-yeast interactions revealed by aromatic profile analysis of Sauvignon Blanc wine fermented by single or co-culture of non-*Saccharomyces* and *Saccharomyces* yeasts. *Food Microbiol.* 32 243–253. 10.1016/j.fm.2012.06.006 22986187

[B59] Saharan R. K. Kanwal S. Sharma S. C. (2010). Role of glutathione in ethanol stress tolerance in yeast *Pachysolen tannophilus*. *Biochem. Biophys. Res. Commun.* 397 307–310. 10.1016/j.bbrc.2010.05.107 20510676

[B60] Saini P. Beniwal A. Kokkiligadda A. Vij S. (2018). Response and tolerance of yeast to changing environmental stress during ethanol fermentation. *Process Biochem.* 72 1–12. 10.1016/j.procbio.2018.07.001

[B61] Schuller D. Casal M. (2005). The use of genetically modified *Saccharomyces cerevisiae* strains in the wine industry. *Appl. Microbiol. Biotechnol.* 68 292–304. 10.1007/s00253-005-1994-2 15856224

[B62] Snoek T. Verstrepen K. J. Voordeckers K. (2016). How do yeast cells become tolerant to high ethanol concentrations? *Curr. Genet.* 62 475–480. 10.1007/s00294-015-0561-3 26758993

[B63] Stanley D. Bandara A. Fraser S. Chambers P. J. Stanley G. A. (2010a). The ethanol stress response and ethanol tolerance of *Saccharomyces cerevisiae*. *J. Appl. Microbiol.* 109 13–24. 10.1111/j.1364-5072.2009.04657.x 20070446

[B64] Stanley D. Fraser S. Chambers P. J. Rogers P. Stanley G. A. (2010b). Generation and characterisation of stable ethanol-tolerant mutants of *Saccharomyces cerevisiae*. *J. Industr. Microbiol. Biotechnol.* 37 139–149. 10.1007/s10295-009-0655-3 19902282

[B65] Sun Y. Zhang T. Lü H. Yu Z. Li X. (2016). Effect of added sulphur dioxide levels on the fermentation characteristics of strawberry wine. *J. Instit. Brew.* 122 446–451. 10.1002/jib.342

[B66] Taillandier P. Lai Q. P. Julien-Ortiz A. Brandam C. (2014). Interactions between *Torulaspora delbrueckii* and *Saccharomyces cerevisiae* in wine fermentation: influence of inoculation and nitrogen content. *World J. Microbiol. Biotechnol.* 30 1959–1967. 10.1007/s11274-014-1618-z 24500666

[B67] Ugliano M. Henschke P. A. (2009). in *Wine Chemistry and Biochemistry*, eds Moreno-Arribas M. V. Polo M. C. (New York, NY: Springer).

[B68] van Breda V. Jolly N. P. Booyse M. van Wyk J. (2018). *Torulaspora delbrueckii* yeast strains for small-scale Chenin blanc and pinotage vinifications. *S. Afric. J. Enol. Vitic.* 39:1652 10.21548/39-1-1652

[B69] Voordeckers K. Kominek J. Das A. Espinosa-Cantú A. De Maeyer D. Arslan A. (2015). Adaptation to high ethanol reveals complex evolutionary pathways. *PLoS Genet.* 11:e1005635. 10.1371/journal.pgen.1005635 26545090 PMC4636377

