# Supplementary material for: Adaptive Laboratory Evolution of Native Torulaspora delbrueckii YCPUC10 With Enhanced Ethanol Resistance and Evaluation in Co-inoculated Fermentation
Source: Front Microbiol. 2020 Dec 21;11:595023. doi: 10.3389/fmicb.2020.595023 (PMC7779481; doi:10.3389/fmicb.2020.595023)
Supplement: Supplementary Table 1 — Volatile composition (mg/L) of the final wines after alcoholic fermentation. FSc, S. cerevisiae EC1118 pure fermentation; FTd/Sc, co-inoculated fermentation of EC1118 and YCPUC10; FTdF/Sc, co-inoculated fermentation of EC1118 and YCPUC10-F. Mean ± SD (n = 3). Different letters after means represent significant differences among yeast combinations for fermentation in each parameter (LSD, p ≤ 0.05). Aroma compounds that OAV exceeding one are highlighted and those compounds that OAV exceeding 0.1 but <1 are underlined. [file Table_1.docx]

Supplementary Material

Adaptive Laboratory Evolution of Native *Torulaspora delbrueckii* YCPUC10 with Enhanced Ethanol Resistance and Evaluation in Co-inoculated Fermentation

Daniela Catrileo^1^, Andrea Acuña-Fontecilla^1^, Liliana Godoy^1^*

* Correspondence: Liliana Godoy: liliana.godoy@uc.cl

Supplementary Table 1. Volatile composition (mg/L) of the final wines after alcoholic fermentation.

|  | Wines | | | |  |
| --- | --- | --- | --- | --- | --- |
| Aroma Compounds | FSc | FTd/Sc | FTdF/Sc | |  |
| *Acids* |  |  |  | |  |
| 2-Ethylhexanoic acid | 0.0005±0.0002^a^ | 0.0006±0.0001^a^ | 0.0005±0.0001^a^ | |  |
| 2-Methylbutyric acid | 0.0019±0.0003^b^ | 0.0020± 0.0001^ab^ | 0.0022±0.0000^a^ | |  |
| 9-Decenoic acid | 0.0171±0.0014^b^ | 0.0197±0.0012^a^ | 0.0185± 0.0007^ab^ | |  |
| Benzoic acid | 0.0004±0.0001^a^ | 0.0003±0.0001^b^ | 0.0003±0.0000^b^ | |  |
| Butyric acid | 0.0006±0.00000^b^ | 0.0007±0.0000^a^ | 0.0006±0.0001^b^ | |  |
| Decanoic acid | 0.0535±0.0044^a^ | 0.0577±0.0049^a^ | 0.0563±0.0026^a^ | |  |
| Dodecanoic acid | 0.0006±0.0001^a^ | 0.0008±0.0001^a^ | 0.0008±0.0001^a^ | |  |
| Heptanoic acid | 0.0004±0.0001^a^ | 0.0004±0.0001^a^ | 0.0005±0.0001^a^ | |  |
| Hexanoic acid | 0.0261±0.0024^b^ | 0.0330±0.0030^a^ | 0.0304± 0.0028^ab^ | |  |
| Nonanoic acid | 0.0017±0.0002^b^ | 0.0026±0.0005^a^ | 0.0025±0.0004^a^ | |  |
| Octanoic acid | 0.0834±0.0064^b^ | 0.0926±0.0013^a^ | 0.0913±0.0061^ab^ | |  |
| **Total acids** | 0.1861±0.0139^b^ | 0.2103±0.0034^a^ | 0.2037±0.0088^a^ | |  |
| *Alcohols* |  |  |  | |  |
| 1-Butanol | 0.0003±0.0000^a^ | 0.0003±0.00000^a^ | 0.0003±0.0000^a^ | |  |
| 1-Decanol | 0.0003±0.00000^a^ | 0.0003±0.0000^a^ | 0.0003±0.0000^a^ | |  |
| 1-Dodecanol | 0.0004±0.0000^ab^ | 0.0004±0.00001^b^ | 0.0005±0.0000^a^ | |  |
| 1-Heptanol | 0.0022±0.0002^a^ | 0.0023±0.0002^a^ | 0.0020±0.0003^a^ | |  |
| 1-Nonanol | 0.0006±0.0000^b^ | 0.0007±0.0000^a^ | 0.0006±0.0000^b^ | |  |
| 1-Octanol | 0.0012±0.0000^b^ | 0.0014±0.0001^a^ | 0.0013±0.0001^ab^ | |  |
| 1-Octen-3-ol | 0.0004±0.0000^a^ | 0.0005±0.0001^a^ | 0.0005±0.0000^a^ | |  |
| 1-Undecanol | 0.0001±0.0000^a^ | 0.0001±0.0000^a^ | 0.0001±0.0000^a^ | |  |
| 2-Ethylhexanol | 0.1784±0.0051^b^ | 0.1824±0.0042^b^ | 0.2059±0.0200^a^ | |  |
| 2-Methyl-1-butanol | 0.1432±0.0143^a^ | 0.1486±0.0061^a^ | 0.1550±0.0040^a^ | |  |
| 2-Phenylethanol | 0.2501±0.00218^b^ | 0.2835±0.0048^a^ | 0.2876±0.0177^a^ | |  |
| 3-Ethoxy-1-propanol | 0.0009±0.00001^a^ | 0.0009±0.0001^a^ | 0.0009±0.0000^a^ | |  |
| Isoamyl alcohol | 0.2076±0.0110^a^ | 0.2075±0.0104^a^ | 0.2179±0.0071^a^ | |  |
| 3-Methyl-1-pentanol | 0.0014±0.0001^b^ | 0.0017±0.0001^a^ | 0.0018±0.0002^a^ | |  |
| 4-Methyl-1-pentanol | 0.0004±0.0000^a^ | 0.0004±0.0000^a^ | 0.0004±0.0000^a^ | |  |
| Benzyl alcohol | 0.0002±0.0000^a^ | 0.0002±0.0000^a^ | 0.0002±0.0000^a^ | |  |
| Isobutyl alcohol | 0.0064±0.0008^a^ | 0.0058±0.0008^a^ | 0.0066±0.0009^a^ | |  |
| **Total Alcohols** | 0.7941±0.0464^b^ | 0.8367±0.0102^ab^ | | 0.8818±0.0353^a^ | |
| *Aldehydes* |  |  |  | |  |
| Acetaldehyde | 0.0016±0.0001^a^ | 0.0017±0.0004^a^ | 0.0018±0.0004^a^ | |  |
| Decanal | 0.0003±0.0000^a^ | 0.0003±0.0001^a^ | 0.0002±0.0000^a^ | |  |
| Dodecanal | 0.0028±0.0004^a^ | 0.0028±0.0008^a^ | 0.0028±0.0003^a^ | |  |
| Hexanal | 0.0001±0.0000^a^ | 0.0001±0.0000^a^ | 0.0001±0.0000^a^ | |  |
| Mesitaldehyde | 0.0005±0.0001^b^ | 0.0006±0.0001^a^ | 0.0006±0.0001^a^ | |  |
| Nonanal | 0.0010±0.0001^a^ | 0.0011±0.0002^a^ | 0.0012±0.0001^a^ | |  |
| Octanal | 0.0001±0.0000^a^ | 0.0001±0.0000^a^ | 0.0001±0.0000^a^ | |  |
| Tetradecanal | 0.0001±0.0000^a^ | 0.0002±0.0000^a^ | 0.0002±0.00001^a^ | |  |
| **Total Aldehydes** | 0.0065±0.0004^a^ | 0.0067±0.0013^a^ | 0.0071±0.0007^a^ | |  |
| *C6 Compounds* |  |  |  | |  |
| 1-Hexanol | 0.0205±0.0005^a^ | 0.0209±0.0008^a^ | 0.0210±0.0004^a^ | |  |
| E-3-Hexenol | 0.0001±0.0000^a^ | 0.0001±0.0000^a^ | 0.0001±0.0000^a^ | |  |
| Z-3-Hexenol | 0.0001±0.0000^a^ | 0.0001±0.0000^a^ | 0.0001±0.0000^a^ | |  |
| **Total C6 Compounds** | 0.0208±0.0006^a^ | 0.0211±0.0008^a^ | 0.0212±0.0004^a^ | |  |
| *Esters* |  |  |  | |  |
| Ethyl 2-hexenoate | 0.0001±0.0000^a^ | 0.0001±0.0000^a^ | 0.0001±0.0000^a^ | |  |
| Ethyl 2-methylbutyrate | 0.0001±0.0000^a^ | 0.0003±0.0002^a^ | 0.0002±0.0001^a^ | |  |
| Ethyl 4-hydroxybutyrate | 0.0003±0.0000^a^ | 0.0003±0.0000^a^ | 0.0003±0.0000^a^ | |  |
| Ethyl 9-decenoate | 0.0075±0.0007^c^ | 0.0123±0.0002^a^ | 0.0112±0.0004^b^ | |  |
| Ethyl acetate | 0.0327±0.0011^b^ | 0.0365±0.0026^a^ | 0.0373±0.0014^a^ | |  |
| Ethyl butyrate | 0.0023±0.0000^b^ | 0.0027±0.0001^a^ | 0.0027±0.0000^a^ | |  |
| Ethyl carbonate | 0.0001±0.0000^a^ | 0.0002±0.0002^a^ | 0.0001±0.0001^a^ | |  |
| Ethyl cinnamate | 0.0001±0.0000^a^ | 0.0001±0.0000^a^ | 0.0001±0.0000^a^ | |  |
| Ethyl decanoate | 0.0124±0.0007^b^ | 0.0202±0.0031^a^ | 0.0215±0.0010^a^ | |  |
| Ethyl dodecanoate | 0.0009±0.0003^b^ | 0.0019±0.0004^a^ | 0.0024±0.0006^a^ | |  |
| Ethyl heptanoate | 0.0001±0.0000^a^ | 0.0001±0.0000^a^ | 0.0001±0.0000^a^ | |  |
| Ethyl hexanoate | 0.0262±0.0038^b^ | 0.0314±0.0009^a^ | 0.0289±0.0021^ab^ | |  |
| Ethyl isovalerate | 0.0001±0.0000^a^ | 0.0001±0.0000^a^ | 0.0001±0.0000^a^ | |  |
| Ethyl lactate | 0.0005±0.0001^a^ | 0.0005±0.0001^a^ | 0.0006±0.0001^a^ | |  |
| Ethyl nonanoate | 0.0001±0.0000^b^ | 0.0002±0.0001^a^ | 0.0003±0.0000^a^ | |  |
| Ethyl octanoate | 0.0236±0.0047^c^ | 0.0378±0.0039^a^ | 0.0294±0.0005^b^ | |  |
| Ethyl phenylacetate | 0.0005±0.0001^a^ | 0.0005±0.0000^a^ | 0.0005±0.0000^a^ | |  |
| Ethyl propanoate | 0.0018±0.0004^a^ | 0.0020±0.0001^a^ | 0.0021±0.0000^a^ | |  |
| Hexyl acetate | 0.0040±0.0006^a^ | 0.0046±0.0008^a^ | 0.0041±0.0008^a^ | |  |
| Isoamyl acetate | **0.0405±0.0016^a^** | **0.0409±0.0008^a^** | **0.0418±0.0028^a^** | |  |
| Isobornyl acetate | 0.0000±0.0000^a^ | 0.0000±0.0000^a^ | 0.0000±0.0000^a^ | |  |
| Isobutyl acetate | 0.0001±0.0000^a^ | 0.0001±0.0000^a^ | 0.0001±0.0000^a^ | |  |
| Methyl benzoate | 0.0005±0.0001^a^ | 0.0006±0.0000^a^ | 0.0006±0.0000^a^ | |  |
| Octyl acetate | 0.0014±0.0004^a^ | 0.0016±0.0003^a^ | 0.0015±0.0001^a^ | |  |
| Phenethyl acetate | 0.0252±0.0018^b^ | 0.0310±0.0009^a^ | 0.0298±0.0014^a^ | |  |
| **Total Esters** | 0.1810±0.0071^b^ | 0.2260±0.0063^a^ | 0.2158±0.0074^a^ | |  |
| *Ketones* |  |  |  | |  |
| 2,6-Di-tert-butyl-4-hydroxy-4-methyl-2.5-cyclohexadien-1-one | 0.0005±0.0000^a^ | 0.0005±0.0000^a^ | 0.0005±0.0000^a^ | |  |
| 2-Nonanone | 0.0005±0.0001^a^ | 0.0006±0.0001^a^ | 0.0005±0.0001^a^ | |  |
| 2-Octanone | 0.0003±0.0000^a^ | 0.0003±0.0000^a^ | 0.0004±0.0000^a^ | |  |
| 5-Nonanone | 0.0003±0.0000^a^ | 0.0003±0.0000^a^ | 0.0003±0.0000^a^ | |  |
| **Total Ketones** | 0.0016±0.0001^a^ | 0.0017±0.0001^a^ | 0.0017±0.0001^a^ | |  |
| *Norisoprenoids* |  |  |  | |  |
| 1.1.6-Trimethyl-1.2-dihydronaphthalene (TDN) | 0.0002±0.0001^b^ | 0.0002±0.0001^ab^ | 0.0003±0.0001^a^ | |  |
| 𝛃-Damascenone | **0.0071±0.0010^a^** | **0.0080±0.0012^a^** | **0.0083±0.0007^a^** | |  |
| Vitispirane isomer 1 | 0.0002±0.0001^a^ | 0.0002±0.0001^a^ | 0.0003±0.0001^a^ | |  |
| Vitispirane isomer 2 | 0.0003±0.0001^a^ | 0.0004±0.0002^a^ | 0.0005±0.0001^a^ | |  |
| **Total Norisoprenoids** | 0.0077±0.0012^a^ | 0.0088±0.0015^a^ | 0.0094±0.0009^a^ | |  |
| *Phenolic Derivatives* |  |  |  | |  |
| 2.4-Di-tert-butylphenol | 0.0376±0.0105^a^ | 0.0408±0.0058^a^ | 0.0471±0.0076^a^ | |  |
| 4-Vinylguaiacol | 0.0024±0.0002^b^ | 0.0030±0.0002^a^ | 0.0030±0.0003^a^ | |  |
| 4-Vinylphenol | 0.0029±0.0003^b^ | 0.0034±0.0001^a^ | 0.0034±0.0003^a^ | |  |
| Phenol | 0.0002±0.0000^a^ | 0.0002±0.0001^a^ | 0.0002±0.0000^a^ | |  |
| Vianol | 0.0022±0.0025^a^ | 0.0035±0.0043^a^ | 0.0038±0.0036^a^ | |  |
| **Total Phenolic Derivatives** | 0.0458±0.0084^b^ | 0.0518±0.0011^ab^ | 0.0575±0.0039^a^ | |  |
| *Sulfur Compounds* |  |  |  | |  |
| Dimethyl sulfide | 0.0000±0.0000^a^ | 0.0000±0.0000^a^ | 0.0000±0.0000^a^ | |  |
| Methionol | 0.0007±0.0002^b^ | 0.0009±0.0000^a^ | 0.0010±0.0001^a^ | |  |
| **Total Sulfur Compounds** | 0.0008±0.0002^b^ | 0.0009±0.0000^a^ | 0.0010±0.0001^a^ | |  |
| *Terpenes* |  |  |  | |  |
| a-Curcumene | 0.0001±0.0000^a^ | 0.0001±0.0000^a^ | 0.0001±0.0000^a^ | |  |
| a-Farnesene | 0.0001±0.0000^a^ | 0.0001±0.0000^a^ | 0.0001±0.0000^a^ | |  |
| a-Terpinene | 0.0001±0.0000^a^ | 0.0001±0.0000^a^ | 0.0001±0.0000^a^ | |  |
| a-Terpineol | 0.0015±0.0002^a^ | 0.0017±0.0003^a^ | 0.0019±0.0002^a^ | |  |
| a-Terpinolene | 0.0004±0.0001^a^ | 0.0005±0.0001^a^ | 0.0006±0.0001^a^ | |  |
| b-Myrcene | 0.0008±0.0001^a^ | 0.0008±0.0001^a^ | 0.0008±0.0001^a^ | |  |
| Citronellol | 0.0005±0.0000^a^ | 0.0005±0.0000^a^ | 0.0005±0.0000^a^ | |  |
| E-b-Ocimene | 0.0004±0.0001^a^ | 0.0005±0.0000^a^ | 0.0005±0.0000^a^ | |  |
| Geraniol | 0.0001±0.0000^a^ | 0.0001±0.0000^a^ | 0.0001±0.0000^a^ | |  |
| g-Terpinene | 0.0001±0.0000^a^ | 0.0001±0.0000^a^ | 0.0002±0.0000^a^ | |  |
| Hotrienol | 0.0012±0.0002^b^ | 0.0014±0.0002^ab^ | 0.0016±0.0001^a^ | |  |
| Limonene | 0.0004±0.0001^a^ | 0.0004±0.0001^a^ | 0.0005±0.0000^a^ | |  |
| Linalool | 0.0037±0.0003^a^ | 0.0039±0.0003^a^ | 0.0040±0.0003^a^ | |  |
| Nerol | 0.0000±0.0000^a^ | 0.0000±0.0000^a^ | 0.0000±0.0000^a^ | |  |
| Nerol oxide | 0.0002±0.0000^b^ | 0.0003±0.0001^ab^ | 0.0003±0.0001^a^ | |  |
| Nerolidol | 0.0002±0.0000^b^ | 0.0003±0.0000^a^ | 0.0003±0.0001^ab^ | |  |
| p-Cymene | 0.0002±0.0000^a^ | 0.0002±0.0001^a^ | 0.0002±0.0000^a^ | |  |
| Z-b-Ocimene | 0.0003±0.0000^a^ | 0.0003±0.0000^a^ | 0.0003±0.0000^a^ | |  |
| Z-Linalool oxide | 0.0000±0.0000^a^ | 0.0000±0.0000^a^ | 0.0000±0.0000^a^ | |  |
| **Total Terpenes** | 0.0103±0.0012^a^ | 0.0112±0,0014^a^ | 0.0121±0.0009^a^ | |  |
|  |  |  |  | |  |
| **Total general** | **1.2547**^b^ | **1.3753**^a^ | **1.4113**^a^ | |  |

FSc, *S. cerevisiae* EC1118 pure fermentation; FTd/Sc, co-inoculated fermentation of EC1118 and YCPUC10; FTdF/Sc, co-inoculated fermentation of EC1118 and YCPUC10-F.

Mean ± SD (n = 3). Different letters after means represent significant differences among yeast combinations for fermentation in each parameter (LSD, p ≤ 0.05). Aroma compounds that OAV exceeding 1 are highlighted and those compounds that OAV exceeding 0.1 but <1 are underlined.
